# The hyperexcitability of dentate granule neurons in organotypic hippocampal slice cultures is due to reorganization of synaptic inputs in vitro

**DOI:** 10.14814/phy2.12889

**Published:** 2016-10-05

**Authors:** Charlie J. Gilbride

**Affiliations:** ^1^ Depatment of Neuroscience, Physiology and PharmacologyUniversity College LondonLondonUK

**Keywords:** Confocal microscopy, electrophysiology, organotypic slice culture, synaptic plasticity

## Abstract

Organotypic hippocampal slice cultures (OHSCs) provide the experimental flexibility of cell culture while leaving much of the natural neuronal connectivity intact. Previously, it was shown that the functional and morphological features of CA1 pyramidal neurons in OHSCs resemble, to a surprising extent, those of CA1 neurons in the acute brain slice preparation. However, the extent to which the characteristics of other principle hippocampal neurons change or are preserved in cultured slices remains to be determined. In the present study, I initially sought to understand whether and how the synaptic inputs and morphology of cultured dentate granule neurons (GCs) differ from GCs that have developed in vivo. To this end, I compared GCs in OHSCs and GCs in acute slices at two equivalent developmental time points (P14 vs. DIV7 and P21 vs. DIV21). The findings suggest that there is considerable reorganization of synaptic input to the organotypic GCs, such that these cells are more susceptible to hyperexcitation than GCs in acute slices after 3 weeks. It appears that this hyperexcitability emerges through an increase in the proportion of mature synapses at proximal dendritic sites and is accompanied by an increase in inhibitory neuron activity. These alterations appear to arise in a coordinated manner such that the substantial increase in excitatory synaptic drive received by the DIV21 GCs in OHSCs remains local and is not translated into excessive output possibly leading to damage or major morphological alterations of downstream pyramidal neurons.

## Introduction

Organotypic hippocampal slice culture (OHSC) is a valuable tool that enables investigators to carry out studies where in vivo experiments are impractical, but where it is desirable to preserve the neuronal connectivity found in vivo. The slice culture system also enables investigators to gain a better assessment of an experimental strategy before beginning time‐consuming in vivo experiments. In each case, the OHSC system is chosen primarily because it is believed to preserve the connectivity of the trisynaptic hippocampal circuit.

The hippocampus is well established as being crucial for encoding new memories in primates and rodents (Bird and Burgess 2008; Neves et al. 2008). With a neuronal network that is heavily influenced by environmental experiences, one could reasonably expect development of hippocampal neurons maintained in slice culture to be radically different from development of the same cell type inside the living animal. However, De Simoni et al. (2003) found surprisingly few differences between CA1 pyramidal cells that had developed in OHSCs (at DIV7, DIV14, and DIV21) and pyramidal cells that had developed normally in the living animal (at P14, P17, and P21). De Simoni et al. (2003) compared miniature synaptic currents, dendritic complexity, spine density, and spine morphology of CA1 pyramidal neurons in acute slices and OHSC preparations. Organotypic CA1 neurons were found to be similar to acute CA1 neurons across most of the parameters measured. Even in measures where organotypic and acute CA1 neurons differed, their change over time in slice culture was found to be proportionally similar to the change occurring in vivo.

In this study, similar experiments were conducted in order to increase understanding of dentate granule cell (GC) development in organotypic slice cultures relative to age‐equivalent acute slices. In this way, the work of De Simoni et al. (2003) has been expanded upon to include the other major cell type of the trisynaptic circuit of the hippocampus – the granule cell of the dentate gyrus.

The results show a much higher frequency of action potential‐driven spontaneous excitatory currents in granule cells (GCs) of 3‐week‐old organotypic slices relative to GCs of acute slices conferring a greater susceptibility to hyperexcitation in the cultured GCs after 3 weeks. The morphological findings indicate that there is an increase in spine density along proximal dendrites of the cultured GCs relative to the GCs of acute slices, which probably underlies the hyperexcitability of GCs maintained in vitro. The results also suggest that the total spine number is likely to be similar in both acute and organotypic GCs after 3 weeks. I propose that the hyperexcitability in organotypic slices is triggered not by hyperconnectivity, but by an increased density of active spines closer to the soma. This increase in excitatory drive of the GCs appears to be balanced by an increase in the activity of local inhibitory interneurons, which probably limits depolarization and output of the GCs.

## Methods

### Animals

Sprague Dawley rats were used in all experiments, which were conducted in accordance with UK Home Office regulations and National Ethics Committee guidelines. Slices were taken from 5‐postnatal‐day‐old (P5) male rats for organotypic cultures and from P14 and P21 male rats for acute slice preparations.

### Preparation of organotypic slice cultures

For the organotypic cultures, sagittal hippocampal brain slices were taken from P5 rats using the interface method (Stoppini et al. 1991; De Simoni and Yu 2006). In brief, the cultures were prepared under sterile conditions in a flow cabinet. Following decapitation, the brain was quickly removed, placed in ice‐cold slicing medium (Earle's balanced salt solution [GIBCO BRL] with 25 mmol/L HEPES buffer) and hemisected. Both hemispheres were mounted on a slicing stage with superglue and slices were cut at 300 μm using a vibroslicer. Once all the slices had been extracted, they were placed on a semicircular slip of 0.45 μm filter paper (FHLC membrane, Millipore) at the bottom of a six‐well culture plate and then placed in an incubator at 37°C and 5% CO_2_. Unless otherwise stated, the section of the entorhinal cortex immediately caudodorsal to the dentate gyrus region in the slice was left attached to the hippocampal formation because this is known to reduce granule cell mossy fiber sprouting (Coltman et al. 1995).

Experiments were carried out after 6–8 days in vitro or 20–22 days in vitro, which are abbreviated to DIV7 and DIV21, respectively.

Culture medium was made up of 25% horse serum, 50% minimum essential medium (with Earle's salts, 25 mM HEPES and GlutaMAX‐1), and 25% EBSS. This medium was supplemented with 6.5 mg/mL glucose and 1–2% antibiotic–antimycotic liquid (containing penicillin, streptomycin, and amphotericin B/nystatin). All components of the culture medium were supplied by Invitrogen.

### Preparation of acute slices

Acute slices were prepared as described previously (Edwards et al. 1989). Briefly, P14 or P21 rats were decapitated and the brain rapidly removed and placed in ice‐cold artificial cerebrospinal fluid (ACSF [in mmol/L]: NaCl 125, KCl 2.4, NaHCO_3_ 26, NaH_2_PO_4_ 1.25, glucose 25, CaCl_2_ 2 [0.5 mmol/L for dissection], MgCl_2_ 1 [5 mmol/L for dissection]; bubbled with carbogen). To reduce the rate of cell death most dissections were carried out with high‐magnesium (5 mmol/L)/low‐calcium (0.5 mmol/L) ACSF. The brain was hemisected and a section was cut away by hand that was approximately 100° from the midline surface. The hemispheres were then mounted with superglue on to a slicing stage and 400 μm hippocampal slices were cut with a vibroslicer and transferred to an incubating chamber with circulating ACSF (bubbled with carbogen). The first chamber that the slices were transferred to typically contained high‐magnesium/low‐calcium dissection ACSF to further aid cell survival. After all the slices were collected from the first hemisphere, they were transferred to another chamber containing standard concentrations of magnesium (1 mmol/L) and calcium (2 mmol/L) in the ACSF. The slices were incubated at 35°C for approximately 1 h before the incubator was switched off and the bath was allowed to cool to room temperature.

Pharmacological blockers were applied to the bath solution and given a 5‐min wash‐in period. Tetrodotoxin (TTX, 1 μmol/L; Tocris/Ascent Scientific – now Abcam plc) was used to measure spontaneous miniature activity. SR95531 (Gabazine, 6 μmol/L) was used to block GABA_A_ receptors, enabling the recording of purely excitatory currents or miniature excitatory currents when applied with 1 μmol/L TTX.

### Electrophysiology

#### Whole cell experiments with caesium chloride internal solution

All whole‐cell patch‐clamp recordings of dentate granule neurons were conducted at room temperature, with a holding potential of −70 mV using an Axopatch 200B or Axopatch 1D amplifier (Axon Instruments, Inc.). The electrode resistances ranged between 4 and 6 MΩ and were pulled from borosilicate glass (World Precision Instruments). Series resistance ranged between 10 and 40 MΩ and was not compensated. The seal and access were checked with a 5‐mV test pulse at least every 5 min. The recording was not included in the analysis if the current amplitude in response to the test pulse changed by more than 30%. Caesium chloride internal solution (mmol/L): CsCl 140, HEPES 5, EGTA 10, Mg‐ATP 2 (pH adjusted to 7.2–7.3 with CsOH). Alexa‐594 (Alexa Fluor 594) 0.2 mg/mL was included in the intracellular solution for imaging the cells' morphology after the electrophysiological recording. All electrophysiological recordings were sampled at 10 kHz and filtered at 2 kHz. Data were acquired using the Strathclyde Electrophysiology Data Recorder (WinEDR; v3.0.6).

#### Whole cell experiments with potassium gluconate internal solution

As with the CsCl internal recordings all experiments were carried out at room temperature using an Axopatch 1D amplifier (Axon Instruments, Inc.). A 15‐mV junction potential was compensated before entering cell‐attached mode. For this combination of internal and external solutions the reversal potential was calculated as approximately −62 mV for GABA‐mediated currents and −3 mV for glutamate‐mediated currents. Potassium gluconate internal solution (mmol/L): KGlu 130, NaCl 10, MgCl_2_ 1, EGTA 1, Hepes 10, glucose 25, Mg‐ATP 2 (pH adjusted to 7.2–7.3 with KOH).

Spontaneous currents were analyzed using Strathclyde Whole Cell Analysis Program (WinWCP; v4.1.0) (WinEDR and WinWCP supplied by Dr John Dempster, University of Strathclyde). The detection threshold was set at −3 pA for 5 msec (and +3 pA for 5 msec, for detecting outward currents). The amplitude of the events was measured using WinWCP.

### Imaging and morphological analysis

During recordings of spontaneous synaptic currents, cells were filled with Alexa‐594 added to the internal solution. The cell was then fixed and imaged within 72 h. Slices were fixed in 4% paraformaldehyde plus sucrose solution followed by two washes with PBS. In a small number of cases, the cells were imaged live.

Cells were imaged using an Olympus Fluoview confocal microscope (generously supplied by Olympus, London, UK) with a 60× water immersion objective. Image acquisition was carried out using Olympus Fluoview imaging software. Initially, the complete image of each cell was obtained in 3 μm increments through the *z*‐axis. Dendrite sections were imaged separately with 0.2‐μm *z*‐steps.

Z‐stack images of GC dendrites were analyzed using ImageJ software (National Institute of Health, USA). Spines were identified as stubby, filopodia, or thin/mushroom following the classification system outlined by Harris et al. (1992). The dendrite classifications used in this study are similar to those previously used to describe pyramidal neuron dendrites (e.g., De Simoni et al. 2003).

Dendrite measurements were carried out using the filament tracing software Imaris (7.0) (Bitplane). Before carrying out the analysis, each image was deconvolved in order to correct for light scatter through the *z*‐plane that was present in each image. For the classification of spines, the experimenter was blind to the identity of the dendrites.

### Statistical analysis

**Figure 1:**
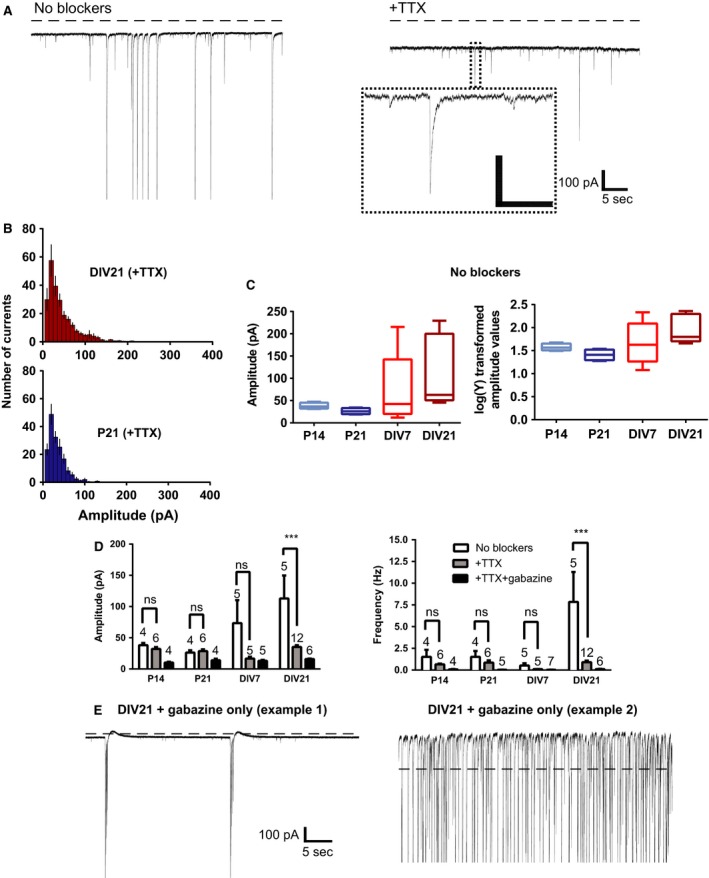
Amplitude and frequency of spontaneous events in acute versus organotypic slices, recorded with a CsCl‐based internal solution. (A) Example trace showing spontaneous postsynaptic currents without blockers (left) and after washing in 1 μmol/L TTX (right) from an organotypic GC at DIV21. Large amplitude currents have been truncated at −900 pA. Dashed line shows the point of zero current offset. Inset: example of a miniature synaptic current. Inset scale: 100 pA, 0.5 sec. (B) Descriptive statistics: Average amplitude frequency distribution for P21 (deep blue) and DIV21 (dark red) miniature currents. There is a very clear positive skew for the distribution of DIV21 miniature currents – necessitating selection of the median rather than the mean as the average current value for a given cell. (C) Descriptive statistics: Untransformed plot of median values (left) shows a clear skew for median amplitude current values from DIV7 (red) and DIV21 (dark red) GCs in the “no blockers” condition. Log transformation of the same data (right) reduces the skew and improves for the condition of the data for Student's *t*‐test. (D) Amplitude and frequency of all spontaneous currents without blockers (white bars), combined excitatory miniature postsynaptic currents (mEPSCs) and inhibitory miniature postsynaptic currents (mIPSCs) (gray bars) and isolated mEPSCs (black bars) from P14, DIV7, P21, and DIV21 GCs. *N*‐values on graphs refer to number of cells; ***P < 0.001. In all cases at least three animals were used to obtain each average, with the following exceptions: For the DIV7 and P21 “no blockers” data, two animals were used. (E) Two example traces from DIV21 GCs. Complete GABAA receptor blockade with gabazine induced rhythmic bursts of activity or high‐frequency epileptiform activity (four cells, two animals). Some currents that were in the nanoampere range appear truncated.

Calculations were computed using Graphpad Prism (Graphpad Software Inc., San Diego). Data presented in Figure 1 were log‐transformed (*Y*_t_ = log(*Y*)) to reduce the data skew and statistical analyses were carried out on the transformed data. Untransformed data are presented in the graphs. Two‐way ANOVAs were used to examine differences between multiple groups and *t*‐tests, corrected for multiple comparisons (Bonferroni post hoc analyses), and were used to test for significant differences between specific groups. Where appropriate, Student's *t*‐tests were used to test for significant differences between two groups. Results are reported as mean ± SEM. Error bars represent standard error of the mean (SEM).

Amplitude distributions for spontaneous synaptic currents (inset, Fig. 1A) have a strong positive skew, both before and, in some cases, after TTX application (Frerking et al. 1995; Wojcik et al. 2004; see Fig. 1B) making the mean current amplitude for individual cells an inappropriate choice as a representative current value. Therefore, for the data presented in Figures 1 and 3, the median current amplitude was calculated for each trace and the mean of these values was used in plotting the data. Student's *t*‐test was applied to means of log‐transformed median values.

## Results

Whole‐cell voltage‐clamp recordings of GCs were carried out in acute and organotypic slices. Spontaneous action potential‐mediated synaptic currents and miniature currents were measured. TTX (1 μmol/L) did not alter the amplitude (Fig. 1D, P14: 38.1 ± 3.5 pA [no blockers] vs. 32.11 ± 3 pA [+TTX]; P21: 26.3 ± 3.6 pA [no blockers] vs. 28.7 ± 2.8 pA [+TTX]) or frequency (Fig. 1D, P14: 1.5 ± 0.8 Hz [no blockers] vs. 0.7 ± 0.1 Hz [+TTX]; P21: 1.5 ± 0.7 Hz [no blockers] vs. 0.9 ± 0.3 Hz [+TTX]) of spontaneous synaptic currents in acute GCs, which implies that there is very little action potential‐driven transmitter release onto these cells in the acute brain slice preparation. On the other hand, the amplitude of spontaneous synaptic currents recorded in DIV21 GCs from organotypic slices was significantly smaller in the presence of TTX compared to the no blockers condition (Fig. 1D, DIV21: 112.8 ± 36.9 pA vs. +TTX, 35.2 ± 2.9 pA, *P *<* *0.001). In many cases, spontaneous currents in the DIV21 “no blockers” condition had numerous saturating currents in the nanoampere range (Fig. 1A left trace). These currents could not contribute to the average current amplitude estimate, so the calculated average for the DIV21 “no blockers” condition is likely to be an underestimate. In addition, the frequency of spontaneous synaptic currents was significantly decreased by the addition of TTX at DIV21 in organotypic slices (Fig. 1D, DIV21: no blockers 7.8 ± 3.5 Hz vs. +TTX, 0.9 ± 0.2 Hz, *P *<* *0.001). Together, these data suggest GCs in organotypic slices develop action potential‐mediated synaptic input after 3 weeks in vitro, while the action potential‐mediated input to GCs in acute slices is very limited or nonexistent.

The absence of action potential‐mediated synaptic activity in acute slices is most likely accounted for by the destruction of the perforant path inputs during slice preparation. Denervation of the GCs probably happens to a similar extent in both the acute slice and OHSC preparation, but with new excitatory synapse formation or regeneration of destroyed efferents taking place over the course of 3 weeks in vitro. Alternatively, the spontaneous action potential‐mediated currents evident in DIV21 GCs could be driven by local inhibitory interneurons present in the granule cell layer of the dentate gyrus (Andersen 2007).

If excitatory inputs to the GCs are formed in DIV21 OHSCs, then cultured GCs will continue to show excitatory activity when inhibitory inputs are suppressed.

When spontaneous currents were recorded in the presence of 6 μmol/L gabazine, large, multisynaptic events were regularly observed in four DIV21 GCs (Fig. 1E). One of the four DIV21 GCs showed rhythmic bursts of large saturating currents (Fig. 1E, left). Three of the four recordings from the DIV21 cells displayed epileptic activity characterized by high‐frequency, saturating currents (Fig. 1E, right). Together, these results show that the DIV21 GCs receive excitatory, action potential‐driven input from regenerated or augmented axons.

So far, the data indicate that organotypic GCs detect action potential‐mediated synaptic currents, whereas GCs in the acute slice preparation detect only quantal release (Fig. 1D). It is likely that local inhibitory interneurons drive some of the action potential‐mediated synaptic currents that were recorded. To elucidate the relative proportion of excitatory versus inhibitory spontaneous synaptic currents in cultured and acute GCs, the internal solution was changed to a potassium gluconate‐based variant. Bath application of gabazine, in the absence of TTX, would induce epileptiform activity, so I segregated the excitatory and inhibitory currents by changing the command potential to the calculated reversal potential for GABA‐mediated currents (−62 mV) or glutamate‐mediated currents (−3 mV).

**Figure 2:**
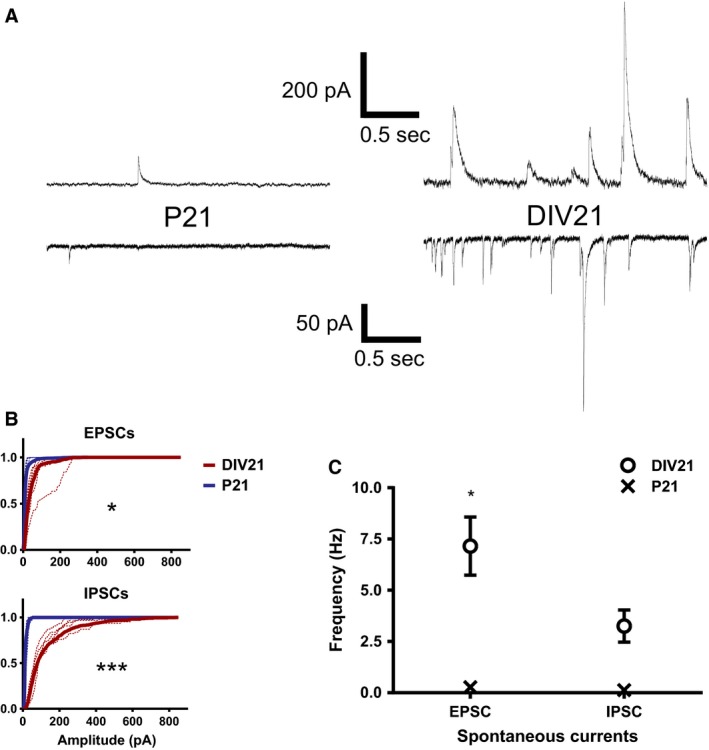
Isolation and recording of spontaneous excitatory postsynaptic currents (EPSCs) and inhibitory postsynaptic currents (IPSCs) in the same GC. (A) Example traces showing inhibitory (outward) currents and excitatory (inward) currents recorded in P21 (left) and DIV21 (right) GCs. Inhibitory and excitatory currents were isolated by adjusting the command potential to the calculated reversal potential for glutamate‐ (−3 mV) or GABA‐ (−62 mV) mediated currents. (B) Cumulative amplitude distributions for EPSCs (above) and IPSCs (below) recorded from P21 (blue) and DIV21 (red) GCs. Solid lines (mean averages) are overlaid with the results from individual cells (dashed lines). Statistical analyses were carried out using repeated‐measures ANOVA. Averages were calculated the same way as for the amplitude data presented in Figure 1: median averages were calculated from individual cells and mean averages calculated from these median averages. (C) The mean frequency of EPSCs and IPSCs. The spread of the P21 data was very small so the error bars are obscured by the symbol (X). Applies to both (B) and (C): Results for Bonferroni post‐hoc tests are indicated on the graphs: *P < 0.05, ***P < 0.001. Applies to both (B) and (C): P21 (*n* = 4 cells/2 animals); DIV21 (*n* = 5 cells/2 animals).

Consistent with the data presented in Figure 1, the frequency of excitatory events was much greater in the DIV21 GCs compared to the P21 cells (Fig. 2C, DIV21: 7.2 ± 1.4 Hz vs. P21: 0.3 ± 0.1 Hz, *P *<* *0.05). Furthermore, the average amplitudes of the excitatory and inhibitory spontaneous currents were significantly larger in the DIV21 GCs (Fig. 2B, EPSCs: 39.7 ± 11.0 pA; IPSCs: 72.4 ± 4.3 pA) compared to the P21 GCs (Fig. 2B, EPSCs: 10.2 ± 0.5 pA, *P *<* *0.05; IPSCs: 14.2 ± 1.9 pA, *P *<* *0.001). These data clearly show excitatory inputs account for a substantial proportion of the spontaneous action potential‐mediated currents, which were elevated in the DIV21 GCs (Fig. 1D). These results also show that an increase in excitatory synaptic drive of the cultured GCs is accompanied by an increase in the amplitude of inhibitory inputs, suggesting that inhibitory synapses of cultured GCs undergo modifications in vitro.

While inhibitory inputs are provided by local interneurons and project to the somatic region of GCs (Soriano and Frotscher 1989; Halasy and Somogyi 1993; Han et al. 1993), the excitatory efferents of the GCs terminate at dendritic spines (Dolorfo and Amaral 1998; Andersen 2007).

**Figure 3:**
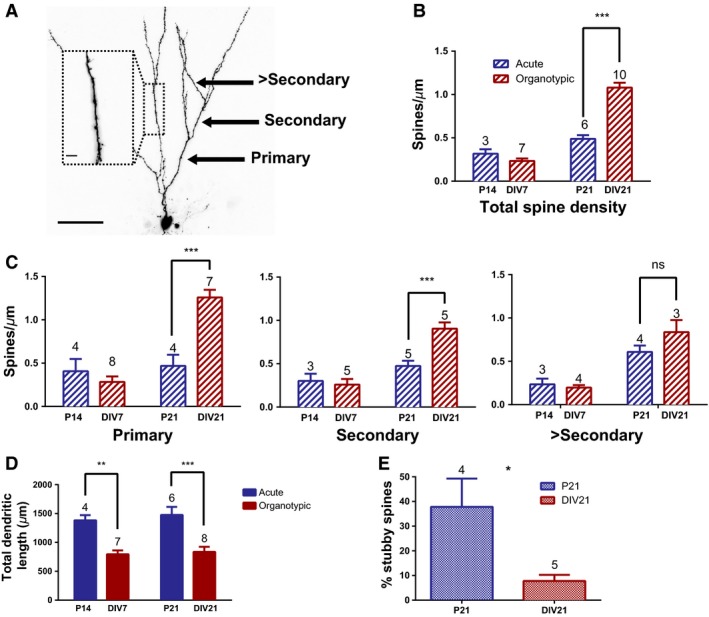
Dendrite classification and spine analysis of GCs from acute and organotypic hippocampal slices. (A) P21 GC filled with Alexa‐594. Dendrite classifications: primary, secondary, or greater than secondary are indicated by the black arrows (scale bar: 50 μm). Inset: example of a 0.2‐μm stack zoom used in the spine analysis, which was acquired across multiple regions of the dendritic arbor in addition to the “whole‐cell” image (scale bar: 5 μm). (B, C) Spine density analysis was carried out on pooled data across the three dendrite classifications (B) and also on isolated primary, secondary, and more distal dendrites from the same data (C) from acute (P14, P21; blue) and organotypic (DIV7, DIV21; red) GCs. (D) The total length of dendrites for GCs in the four groups, measured using the “whole‐cell” images such as the one shown in (A). (E) The proportion of stubby spines along the primary dendrites of P21 and DIV21 GCs. *T*‐tests were used to measure statistical significance *P < 0.05; **P < 0.01; ***P < 0.001. In B, C, D and E the numbers above the graphs are the number of cells included in the analysis. The number of animals used was at least three for all the data shown, except for the DIV7 secondary dendrites in (C) where two were used.

**Figure 4:**
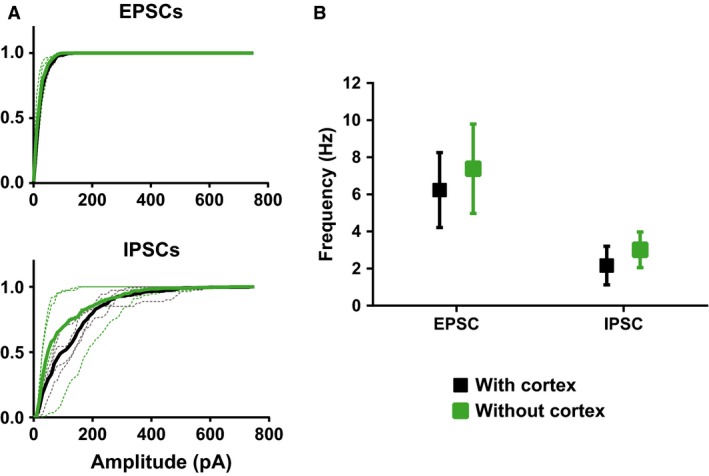
Excitatory and inhibitory spontaneous synaptic currents measured in DIV21 GCs with part of the entorhinal cortex left attached to the slice or completely removed during the slice preparation. (A) Cumulative frequency distributions of the peak amplitudes for excitatory and inhibitory currents. (B) Filled squares represent the mean current frequency of GCs in OHSCs where part of the entorhinal cortex was left attached. Green squares represent the same measurements, but from GCs in OHSC with the cortex completely removed. *N* = 4 cells analysed in the “with” and “without” groups. Two animals were used in these experiments.

To examine changes in the density and morphological type of spines present along the dendrites of acute and organotypic GCs, the cells were filled with Alexa‐594 and imaged by confocal microscopy. Dendrites were classified according to previously established criteria (De Simoni et al. 2003; Fig. 3A). The spine density analysis revealed that the spine density along DIV21 dendrites is double the spine density along P21 dendrites (Fig. 3B, DIV21: 1.1 ± 0.1 spines/μm vs. P21: 0.5 ± 0.04 spines/μm, *P *<* *0.001). However, it should be noted that there is likely to be no change in overall spine number occurring in the OHSC preparation because total dendrite length in DIV21 GCs was almost half that of the P21 GCs (Fig. 3D, DIV21: 836.6 ± 87.2 μm vs. P21: 1476 ± 139.3 μm). The main categories of spine types that have been used in previous publications (Harris et al. 1992; De Simoni et al. 2003) to classify spines are thin, mushroom, filopodia, and stubby spines. Stubby spines are distinct from the other types of spine in that they lack any discernible spine neck and the head of the spine appears to be continuous with the parent dendrite. A thin spine neck increases the resistance between spine head and the dendrite, which increases the strength of local depolarization and subsequent Ca^2+^ ion influx to the spine. Thus, a spine neck is thought to confer important computational properties (Yuste 2013) and the absence of a neck is considered to be an indication that a spine is not fully mature (Harris et al. 1992; Bourne and Harris 2011). The proportion of stubby spines, along the primary dendrites of DIV21 GCs, was found to be much lower compared to P21 GCs (Fig. 4E, DIV21: 7.8 ± 2.5% vs. P21: 37.8 ± 11.4%, *P *<* *0.05). Together, the morphological data indicate that the DIV21 GCs undergo considerable synapse remodeling and possess a higher spine density along proximal dendrites compared to P21 GCs. These morphological changes probably underlie the elevated frequency of spontaneous excitatory currents that were detected in DIV21 GCs.

The differences in spine morphology between DIV21 and P21 imply that the elevated amplitude and frequency of spontaneous currents in DIV21 GCs is caused by the emergence of new spines on the proximal dendrites of cultured GCs. One possibility is that regenerated entorhinal projections to the molecular layer of the dentate gyrus trigger GC hyperexcitability by DIV21.

Although during OHSC preparation a segment of the entorhinal cortex was left attached to the hippocampal formation, it is expected that much of the perforant path projection to the dentate gyrus to be destroyed in this OHSC preparation, given the neuroanatomy of the entorhinal‐dentate connectivity (Witter 1993). However, cortical projections can regenerate in vitro when brain slices are prepared from P5 animals (Li et al. 1995, 1996). To test the hypothesis that synchronous excitatory activity is caused by regenerated cortical projections to the dentate gyrus, experiments similar to those described in Figure 2 were carried out. DIV21 GCs in OHSCs, where the entorhinal cortex had been completely removed, were compared with GCs cultured in slices where the dorsal section of the cortex was left attached to the hippocampal formation. If cortical inputs really do regenerate and form synapses along the proximal regions of the GCs to drive excitatory activity by DIV21, then it could be expected that GCs situated in slices without any cortical tissue to have a lower amplitude and frequency of spontaneous EPSCs compared to GCs in slices with part of the cortex left attached to the hippocampal regions. There was no difference between the GCs cultured with the cortical section and those cultured without, in terms of amplitude of spontaneous EPSCs (Fig. 4A, excitatory currents: “with cortex”: 22.1 ± 1.8 pA vs. “without cortex”: 20.8 ± 4.2 pA; inhibitory currents: “with cortex”: 107.8 ± 19.1 pA vs. “without cortex” 80.5 ± 36.8 pA) and frequency (Fig. 4B, excitatory currents: “with cortex”: 6.2 ± 2.0 Hz vs. “without cortex”: 7.4 ± 2.4 Hz; inhibitory currents: “with cortex”: 2.2 ± 1.0 Hz vs. “without cortex” 3.0 ± 1.0 Hz) of spontaneous synaptic currents.

Together, the results suggest that the preparation of OHSCs triggers a cascade of changes in the dentate granule neurons involving both an increased excitatory and inhibitory drive. Excitatory input from the cortex is lost, but is balanced by increased input from another source – most likely, hilar mossy cells (see Discussion). For reasons that are currently unclear, compensatory increases in the excitatory drive closer to the somata seems to require balancing by inhibitory drive from interneurons located in the cell body layer. This balancing of excitatory and inhibitory drive probably contributes to preserving the cells of the slice for long periods under in vitro conditions.

## Discussion

By comparing the spontaneous synaptic currents and morphology of GCs in cultured and acute brain slices, it was found that the excitability of GCs in mature, cultured slices appears to be conferred by a shift in the number of excitatory inputs to more proximal dendrites. The absolute number of spines is similar in both mature brain slice preparations given that the total spine density in DIV21 GCs was double that of P21 GCs, but the length of P21 GC dendrites was almost double that of the DIV21 cells. In addition, the data suggest that it is not regenerated entorhinal cortical inputs that contribute to the increased density of excitatory synapses along the proximal dendrites, which drive the excitatory currents seen in DIV21 GCs.

Initially, spontaneous synaptic currents were examined in acute and organotypic GCs at two different time points. In subsequent experiments it became clear that both excitatory and inhibitory inputs contribute to the elevated amplitude and frequency of synaptic currents that were initially observed. The elevated amplitude and frequency of spontaneous excitatory currents in the DIV21 GCs compared to the P21 GCs can be reasonably explained by a combined effect of deafferentation of the molecular layer in the acute slice caused by the slice preparation procedure and new excitatory synapse formation in the cultured slice preparation by DIV21.

Vlachos et al. (2012) have shown convincingly that cutting the entorhinal cortical afferents to the dentate gyrus, in an entorhinal–hippocampal coculture system, leads to a transient homeostatic increase in the amplitude of mEPSCs in selective regions of the GC dendrites. This increase in the mEPSC amplitude was specific to the more distal dendritic regions of the GCs in the outer molecular layer, which receives input from the entorhinal cortex in vivo. Vlachos and coworkers have proposed that the renormalization of the mEPSC amplitudes arises as the GCs are reinnervated by mossy cell axons – an excitatory neuronal type present in the hilus with axonal projections targeting the inner molecular layer of the dentate gyrus (Vlachos et al. 2012).

The hilus mossy cell is a highly plausible contributor to the heightened excitatory synaptic drive of the DIV21 GCs given that the results show an increased spine density along the proximal dendrites (which largely overlaps with the domain of the inner molecular layer – the target region for mossy cell boutons).

In addition to susceptibility to hyperexcitation, it was found that GCs in this OHSC preparation possess action potential‐mediated inhibitory synaptic currents after 3 weeks in vitro. This property seems to be absent from the GCs in the P21 acute slice preparation. The increase in local inhibitory interneuron activity could be explained as a homeostatic response, which is necessary to balance increased excitatory activity (Karmarkar and Buonomano 2006; Pozo and Goda 2010).

It might be expected that the downstream target cells of GCs – CA3 pyramidal neurons – would be vulnerable to excessive excitatory activity of the GCs unless local interneurons, targeting GCs, increase their synaptic drive to limit excitotoxicity. The dampening of the GCs' output by local interneurons may also reduce the extent of synaptic remodeling in downstream neurons – such as the CA1 neurons, which were reported to be remarkably similar in organotypic and acute slices after 3 weeks of development (De Simoni et al. 2003). Thus, it seems that organotypic GCs undergo extensive remodeling in response to deafferentation, but this remodeling occurs in a controlled and localized manner, which normalizes the output of these neurons and effectively shields neurons further along the trisynaptic hippocampal circuit from similarly dramatic changes in their synaptic and morphological properties.

## Conflict of Interest

None declared.

## Supporting information




**Data S1.** Supplementary Materials 1.

**Data S2.** Supplementary Materials 2.

**Data S3.** Supplementary Materials 3.

**Data S4.** Supplementary Materials 4.
