# Supplementary material for: The hyperexcitability of dentate granule neurons in organotypic hippocampal slice cultures is due to reorganization of synaptic inputs in vitro
Source: Physiol Rep. 2016 Oct 5;4(19):e12889. doi: 10.14814/phy2.12889 (PMC5064129; doi:10.14814/phy2.12889)
Supplement: Supplementary file 5 [file PHY2-4-e12889-s005.docx]

**RawData_Fig1**. Amplitude and frequency data for each cell included in the analysis for Figure 1. The individual currents measured to produce the corresponding median current values along row 6 are supplied below the name for each cell. Each cell is named according to a combination of the initials of the experimenter who acquired the

data (CG – Charlie Gilbride or XW – Xiaowei Wang) and

the date plus a seventh number at the end. This final

number indicates the order of acquisition. CG0911093:

this cell was the third recording acquired on the ninth of

November 2009. Cell names are color-coded if they contributed

to more than one condition. Names appear in

black if currents from this cell contributed to only one

condition. Access resistances measured through the peak

amplitude of the slow capacitance transient and the

beginning and the end of the recording are also supplied.

The amplitude of the membrane capacitance current triggered

by a 5 mV test pulse was estimated by eye to the

nearest 5 pA. Example traces were generated from matlab

files, which were exported from WinEDR files.

**RawData_Fig2**. layout is similar to Figure S1. Excitatory and

inhibitory currents are displayed for DIV21 and P21 GCs.

The cell colored in red was excluded from the ANOVA

because the median current value was an outlier. Traces

in Figure 2A were generated in Graphpad.

**RawData_Fig**3. Total dendrite length and a breakdown of the

different dendritic compartments analyzed for each cell. A

color code aids identification of any one of the three dendrite

classifications: blue – main apical, yellow – primary,

purple – secondary, orange – more than secondary. Main

apical was included in the analysis for Figure 3B, but was

not analyzed as an isolated dendritic subtype. “Dendrite

length” is not a sum of the individual segments displayed.

Dendrite length is derived from tracing the dendrites

viewed in a “whole cell” image using Imaris. N-values for

dendrites, cells, and animals are list in the sheet named

“n-values (spine den. &. . .)” for each dendrite classification

of P14, P21, DIV7, and DIV21 GCs. Spine classifications

for primary dendrites are given in the sheet named

“%spine types.” The n-values for the spine types on primary

dendrites are listed in the sheet marked: “n-values

(%spine types).” Values for the secondary dendrites are

also listed, but these were not used in the final analysis.

**RawData_Fig4**. Same layout as for Figure S2. This sheet contains

peak amplitude and frequency values for excitatory

and inhibitory currents recorded from DIV21 slices plated

with and without entorhinal cortex left attached to the

hippocampal regions.
